# Daring to dream: reactions to tobacco endgame ideas among policy-makers, media and public health practitioners

**DOI:** 10.1186/1471-2458-11-580

**Published:** 2011-07-20

**Authors:** Richard Edwards, Marie Russell, George Thomson, Nick Wilson, Heather Gifford

**Affiliations:** 1 Department of Public Health, University of Otago, Wellington, P.O. Box 7343, Wellington South. New Zealand; 2 Whakauae Research Services, Te Maru o Ruahine Trust, Rm 208, Community House, 66 Karaka St, Whanganui, New Zealand

## Abstract

**Background:**

Tobacco control strategies have mainly targeted reducing demand. Supply-side focused measures, though less familiar, deserve consideration, particularly to achieve 'endgame' tobacco control aims (e.g. achieving close to zero smoking prevalence). We explored attitudes towards supply-side focused 'endgame' tobacco control approaches and how they can be best communicated with senior policymakers, journalists, and public health practitioners.

**Methods:**

We identified five supply-side focused approaches which could potentially lead to the tobacco endgame: two structural models and three discrete actions. The structural models were: (i) a Nicotine Authority to coordinate tobacco control activities and regulate the nicotine/tobacco market for public health aims; and (ii) a Tobacco Supply Agency acting as a monopoly purchaser of tobacco products and controlling the tobacco supply for public health aims. The actions were: (a) allocating progressively reducing tobacco product import quotas (the 'sinking lid') until importation and commercial sale of tobacco products ceased; (b) making tobacco companies responsible for reducing smoking prevalence with stringent financial penalties if targets were missed; and (c) new laws to facilitate litigation against tobacco companies. These approaches were presented as means to achieve a tobacco free New Zealand by 2020 to 19 senior policymakers, journalists, and public health physicians in two focus groups and eight interviews, and their reactions sought.

**Results:**

The tobacco-free vision was widely supported. Participants engaged fully with the proposed tobacco control approaches, which were viewed as interesting or even intriguing. Most supported increasing the focus on supply-side measures. Views differed greatly about the desirability, feasibility and likely effectiveness of each approach. Participants identified a range of potential barriers to implementation and challenges to successfully advocating and communicating these approaches. The current framing of tobacco as a risky but legal commodity was noted as an important potential barrier to implementing endgame approaches.

**Conclusions:**

Endgame tobacco control approaches were considered to be viable policy options. Further policy analysis, research and public discussion are needed to develop endgame approaches. A significant change in the public framing of tobacco may be a prerequisite for implementing endgame solutions.

## Background

Around the world, interventions to reduce smoking prevalence have mostly been introduced incrementally and have mainly focused on trying to reduce demand. Examples include providing support for smoking cessation, health education in schools and through mass media campaigns, and mandated warnings on packaging. Regulation has mostly been limited to controls on marketing, such as restrictions on advertising and sponsorship, and more recently on point of sale tobacco product displays [1]. There has also been substantial regulation in many jurisdictions on where smoking can occur, with restrictions on smoking in indoor public spaces and workplaces now commonplace in developed countries [2,3].

There has been less attention paid to regulation of the nature of tobacco products, or to restricting their supply and availability, other than interventions aimed at minors such as minimum age of purchase [4,5]. A few jurisdictions have regulated the fire safety [6,7], or flavours of tobacco products [8]. More fundamentally, there have been few efforts to address the structure of the supply and distribution system for tobacco products. In most countries this is an unregulated market, in which tobacco companies manufacture and/or import and distribute tobacco products, and retailers sell tobacco products on a commercial basis with the aim of maximising sales and profits [9]. In those countries where the state had a monopoly on tobacco product distribution, they are mostly being transformed through takeover and collaboration with tobacco companies from the developed world. Such monopolies have acted to maximise return to the state rather than in the interests of public health, except possibly unintentionally by being less efficient and aggressive than companies at promoting their tobacco products [10-12].

As a result, compared to other potentially hazardous products such as pharmaceuticals and agricultural poisons like pesticides, there is little or no control of content and design, or of the supply and retailing of tobacco products. This contrasts with other nicotine delivery devices such as nicotine replacement therapies, which are far more rigorously regulated as pharmaceuticals. Hence there is a regulatory imbalance between smoked tobacco and nicotine replacement therapy (NRT) products [13-15], in which the degree of regulation is in inverse relationship to a product's toxicity and harm to population health [16]. In addition, in most countries there is a tension between commercial pressures from companies which profit from tobacco sales, and the public health need to reduce tobacco use to protect health. Because tobacco products are often heavily taxed, there is also a potential tension within governments between tobacco as a source of government revenue, and the social and economic consequences of tobacco use [14,17,18].

There is growing interest internationally in policy solutions that target the nature of the nicotine market. These include balancing the regulatory playing field in favour of non-smoked or non-tobacco nicotine maintenance. One suggested mechanism is to introduce regulatory authorities with sufficient powers to limit commercial activity, and achieve a healthier regulatory balance between smoked tobacco and pharmaceutical or other safer nicotine delivery products [18-23]. More definitively, some authors have argued that the tobacco product production and distribution systems should be changed, for example, through the creation of a not-for-profit distribution agency [24], making the tobacco industry a not-for-profit enterprise [9,25], or through a progressive reduction in the importation and release for sale of smoked tobacco products [26,27].

In parallel with this debate about methods, has been a renewed focus on policy end points. National strategies have often included goals which focus on process indicators (for example numbers of smokers trying to quit) or have set modest, incremental targets for smoking prevalence reduction [28].

Recently some tobacco control organisations and official bodies have advocated more ambitious targets where the aim is to reduce tobacco smoking prevalence to close to zero within the short to medium term. These targets represent a tobacco 'endgame' scenario. For example, in Finland a Tobacco Act was passed in 2010 which aimed to "put an end to the use of tobacco products in Finland", though no mechanism for achieving this was detailed [29]. This Act followed a recommendation from The Cancer Society of Finland that Finland should be wholly smokefree by 2040 [30]. In New Zealand, the Tupeka Kore (tobacco free) vision was launched by a range of concerned NGOs and advocacy groups in 2009. This proposed a target and a series of interventions to achieve close to zero tobacco smoking prevalence by 2020 [31]. Subsequently, the Māori Affairs Parliamentary Select Committee released a report recommending that New Zealand should be smokefree by 2025 [32], and the Government has since affirmed support for this goal [33].

However, the increasing interest in supply-side and endgame strategies has seldom been reflected in the political agenda or national tobacco control strategies. The most dramatic exception is in Bhutan, where the sale of tobacco products was banned in 2004 [34].

The study we report here was carried out in New Zealand where tobacco-related harm remains high. Following substantial declines in the 1970s and 1980s, recent trends have been for a very gradual decline in smoking prevalence [35,36], which remains about 20% among adults, with much higher prevalences among young adults, Māori (above 40% prevalence) and socio-economically disadvantaged groups [37]. Tobacco is not grown commercially in New Zealand and is all imported in raw form (for a single cigarette manufacturing plant) or is imported as manufactured cigarettes. Current regulation greatly limits advertising and sponsorship, requires indoor public areas to be smoke-free, and mandates pictorial health warnings on packs. In addition, increased taxation (price) and social marketing have been used to try to reduce smoking and exposure to secondhand smoke. The cessation system includes a national telephone Quitline, considerable availability of subsidised NRT products and some specific cessation support programmes for high-risk groups.

Preliminary (unpublished) pilot work by the authors in 2008 suggested that radical endgame solutions are poorly understood by the public, media and policy makers, and are difficult to communicate. It was therefore timely to investigate the feasibility and acceptability of these new approaches to tobacco control, in the absence of any body of literature that had probed these issues of understanding and communication for endgames.

The aim of the 'Daring to Dream' study was to the investigate views and understanding of the public, health practitioners, media and policy makers to innovative supply-side and endgame strategies to tobacco control. We report here on phase one of this study in which we explored views about five endgame-orientated tobacco control approaches with public health physicians, media commentators and policy-makers in New Zealand. The second phase of the study involved assessing the reactions of policy-makers, tobacco control and public health practitioners, and smoking and non-smoking members of the public to the communication of a specific endgame strategy. Specifically, in this paper we set out to explore how easily the five endgame approaches were understood, discuss possible communication strategies, and explore participants' reactions to the ideas.

## Methods

From a review of relevant literature and discussions between the team and the project's Advisory Group, we identified five novel tobacco control interventions or approaches. They shared two key characteristics. Firstly, they addressed to some degree the supply-side of tobacco control. Secondly, all were judged to have strong potential to achieve or substantially contribute to achieving the tobacco 'endgame' of close to nil smoking prevalence. An important additional consideration was the feasibility and appropriateness of the interventions in the New Zealand context. For example, mandating a not-for-profit tobacco industry [9,25] in New Zealand was rejected, as nearly all smoked tobacco products in New Zealand are imported.

The five ideas (see table 1) comprised two *structural models *and three *actions*. The *structural models *were overarching changes to the regulatory framework or structure of the tobacco product market. The first was the introduction of a semi-autonomous Tobacco Control Authority with strong regulatory powers [20,21]. The second was the implementation of a semi-autonomous agency (The NZ Tobacco Supply Agency) to act as a monopoly purchaser of tobacco products, and control a not-for-profit supply and distribution system [24,38,39].

**Table 1:** The five endgame approaches as presented to participants

Endgame approach	Key features of model or action
**Model 1: Nicotine Authority**	Regulates the nicotine/tobacco market to achieve public health aims
	Government-owned but independent from government
	Operating costs could be covered from tobacco tax revenues
	Broad range of possible roles and powers, e.g.:
	• Set tobacco tax and retail price levels
	• Control all aspects of tobacco product marketing and design: including retailing, plain packaging, ingredients and additives, nicotine content etc.
	• Require tobacco businesses to disclose all required information to it
	• Fund and commission tobacco control activities such as social marketing campaigns and quit smoking support
	Introduce subsidies for safer nicotine products.

**Model 2: Tobacco Supply Agency**	Non-profit-making independent (but government-owned) agency that controls supply and access to tobacco/nicotine products
	Has public health aims, not-for-profit
	Operating costs could be covered by tobacco tax
	Tobacco manufacturers can only sell products to the Agency
	Agency specifies features such as product packaging, ingredients and additives, and nicotine content
	Agency supplies products to licensed retailers, and sets requirements for their activities, including retail prices
	Agency could also have a broad range of activities like those described for the Nicotine
	Authority (see Model 1 above)
	Fixed life - to end in 2020 or before, when targets met.

**Action 1: Tobacco companies bid for reducing quotas**	Tobacco companies bid for quotas to supply cigarettes and tobacco to New Zealand market
	Quotas reduced progressively e.g. 5% absolute reduction every six months, reducing to zero in 10-15 years.

**Action 2: Penalties for tobacco companies, to promote rapid smoking reductions**	A new law mandates yearly targets for tobacco companies to reduce smoking prevalence and end smoking over a fixed period (e.g. to zero in 10-15 years)
	Severe penalties imposed on tobacco companies ($ many millions) if the targets are not met.

**Action 3: New laws make it easier to take tobacco companies to court - and win**	New legislation or change to existing legislation (e.g. strengthening the New Zealand Fair Trading Act) to make it substantially easier for individuals, groups or the government to take tobacco companies to court for selling a harmful product
	Companies could be prosecuted for things like failing to make cigarettes fire-safe, or for adding chemicals that make tobacco more addictive and cigarettes sweeter-tasting.

The *actions *were more discrete tobacco control interventions with a supply-side focus. The first was a proposal that a 'sinking lid' system of reducing import quotas (10% reduction in absolute terms per year) should be introduced for tobacco products, until importation and commercial sale of tobacco products ceased in 10 years time [27]. The second was to introduce a system in which tobacco companies were made responsible for achieving annual targets for reduction in smoking prevalence and tobacco consumption, with a zero prevalence and consumption target over a fixed period (10-15 years) [40]. Failure to achieve targets would result in stringent penalties for the tobacco companies. The third action was that new legislation should be introduced (or existing legislation amended) to facilitate individual and group-based legal action against the tobacco industry in relation to the harm caused by tobacco products. The actions by British Columbia (joined later by other Canadian provinces) to legislate to enable such litigation is a version of this action [41].

We used in-depth qualitative research methods - including interviews and focus groups - for data collection during May to August 2008. We carried out two focus groups with (i) six public health physicians (Group 1), and (ii) three policy makers and two journalists (Group 2); and in-depth interviews with four policy makers and four journalists. The participants included a mix of senior policy officials from the Ministry of Health, as well as from other sectors of government (e.g. Treasury). We purposively selected participants to gather a diverse range of opinions from a range of key stakeholders in the policy-making process and experienced senior journalists from national news media, of whom only one was a specialist health reporter. We identified participants through consultation within the research team and with the Advisory Group, and via snowballing recruitment [42]. Participants were approached and invited to participate by one of the authors (MR).

We summarised the five approaches in an information sheet provided in advance for the study participants. We sent additional information in the form of key articles about some of the approaches to participants ahead of the focus groups and interviews, if requested.

Participants in an initial focus group with public health physicians suggested that it was important to provide a justification for implementing supply-side and other major tobacco control measures, and to outline a credible vision of what the endgame approaches were seeking to achieve. Therefore, contextual data showing the burden of disease and mortality caused by smoking and the slow decline in smoking prevalence in New Zealand over the previous decade together with an outline of a vision of a tobacco-free future were added to the information sheets for the subsequent interviews and focus group (Group 2). The vision portrayed a tobacco free future in which the next generation of children in New Zealand was protected from tobacco smoking. This was adapted from the existing Tupeka Kore vision [31].

The focus groups were carried out by two of the authors RE and MR. Interviews were carried out by MR, sometimes with additional input from RE. We carried out data collection in a university setting or in individual participants' offices, as convenient for the participant. We used a schedule to guide the content of the interviews and focus groups. Interviews and focus groups were audio-recorded and transcribed.

The transcripts were coded and analysed to identify key themes and sub-themes. We identified themes from those within the interview/focus group schedule, and also new themes arising inductively from participants' statements. One of the authors (MR) prepared a data matrix in which relevant quotes from the interviews and focus groups were collected together under each identified theme and sub-theme. The dominant and divergent or contrary opinions within each theme and sub-theme were identified by MR. Quotes were chosen by MR and RE to illustrate participants' views where they were particularly apt and succinct. The final write up of the results and choice of quotes was back-checked by RE against the data matrix to ensure that it was a valid representation of the findings.

The research was granted ethics approval through the University of Otago ethics approval process (Category B). Participation was voluntary and all participants gave their informed consent to participate and were assured of anonymity.

## Results

### General views on endgame and supply-side ideas for tobacco control

All participants supported increased action against tobacco, and discussed endgame solutions with keen interest.

*"How far we've come - it's the story isn't it? ...and what's the next chapter, and how do we get to the epilogue?" *- Public Health Physician

The vision of a tobacco-free world for New Zealand children by 2020 resonated strongly with research participants who used words like '*compelling'*, *'powerful' *and *'fantastic' *to describe it, though there was also some scepticism expressed whether the timeframe was realistic, and a comment that it should be tested to see if it worked for Māori. The focus on children was mostly seen as a particularly strong point.

" ...points about children being protected from seeing tobacco [smoking] as a normal and benign behaviour and protected from secondhand smoke in all settings would be widely acceptable." - Policy Official

" ... this idea of us older folk having created an environment which is damaging to our kids is very powerful." - Policy Official

There was mostly strong support for exploring innovative supply-side focused tobacco control measures.

*"We have looked at demand... - but we need to look at supply-side and [the]... regulation of that, or management of that in a better way. .... got to get politicians or the community to think about ... stronger supply-side management" *- Policy Official

Participants considered it important in communicating innovative supply-side ideas to provide a clear rationale for supply-side activities, and to include the economic as well as health costs of tobacco. The tobacco industry was generally seen a key barrier to tobacco control, with strong measures needed to counter its influence.

### Views on specific models and actions

All five of the endgame concepts were received favourably by some of the participants, but there was little consensus about the preferred model or action. There was a general sense that supply-side measures would be difficult to achieve politically, and numerous potential barriers were suggested.

### Model 1: Nicotine Authority

Research participants noted that a Nicotine Authority would address the current asymmetry between the industry and consumer. Positive features mentioned included that it would facilitate the introduction of further tobacco control measures (including harm reduction and supply-side focused policies) and would build on the current smoke-free momentum in New Zealand. Another positive feature noted by some participants was that it didn't raise the perceived moral and ethical dilemmas presented by government supply of tobacco products (see Model 2 below). It was mostly seen as a '*sensible*', less radical approach.

One of the possible powers for the authority was stated by the research theme as setting levels of tobacco product taxation. This was a central issue for many participants. Some participants saw the ability to set tax as crucial for the agency to be effective. However, others argued it was unlikely such an Authority would ever be allowed to set tax rates itself; and that it would be politically unacceptable and strategically undesirable for the Authority to control its own income from tax and duty levels.

There was a range of other critical comments. Some participants noted that with a Nicotine Authority the legitimacy of the industry is preserved and the structure of the tobacco product market is left intact. As a result it may have minimal impact, representing only a weak '*slap on the wrist*' for the industry. Others noted that the Authority may provide industry with a negotiating platform, and could be a focus for on-going and costly conflict with the industry.

*"Model 1 strikes me as harm reduction... what can we do without dealing with the fundamental supply system" *- Public Health Physician

*"You leave the beast intact inside your country and it will find a thousand ways to fight ...[that] would be very expensive for the Nicotine Authority" *- Policy Official

Others thought the Authority could result in nicotine addiction being accepted and maintained in the population, and that nicotine would be perceived to be treated, confusingly, as both a medicine *and *a poison.

*"Less desirable than some of the other models - you're accepting nicotine addiction ... A win in public health terms but ... less desirable if perpetuating nicotine addiction." *- Public Health Physician

Finally, some participants had concerns about the effectiveness of a Nicotine Authority in practice. One participant thought, based on experience from other sectors, that the Authority would be subject to constant time-consuming Official Information Act requests, and expensive legal attacks. Another thought that it would be complex to set up, and it would only occur if there was a broad coalition and cross-party political support.

### Model 2: New Zealand Tobacco Supply Agency

The Tobacco Supply Agency was seen by some participants as more of an endgame solution than the Nicotine Authority. Participants identified the Agency as having more powers and multiple functions, including combining demand-side and supply-side activity. Those who viewed this approach positively felt that it was simple and bold, had a clearer supply-side focus, addressed directly the role of the tobacco industry, and accepted that industry was '*fatally flawed*' and had to be removed.

*"Model 2 is much bolder, and much clearer... it ....identifies the industry as the problem ..." *- Policy Official

*"[it] ..seems to more effectively reduce their [tobacco companies'] power base within the country." *- Policy Official

*"You've got to pick your ground and I reckon model two picks much more interesting ground to fight on." *- Policy Official

The most commonly identified barriers were the perceived political and ethical difficulties of the government selling tobacco or nicotine. One policy official feared this could be viewed as retrograde, like "*going back to prohibition"*. Some participants viewed that as *'weird' *or *'dissonant'*; and thought such ethical issues would make it difficult to communicate the idea and gain public acceptance. This would create political difficulties.

*"I feel queasy about the state setting up an agency to peddle something which is inherently harmful" *- Policy Official

*"You are essentially having the government as a dealer of tobacco. I think that would be quite a difficult position for a government to be in... I don't like that idea" - *Journalist

In addition, if the government controlled the supply and sale of tobacco products, there could be perverse incentives for the government to encourage consumption in order to maintain sales and revenue generation. Some participants thought that the Agency could send conflicting messages: such as 'legitimising' a harmful substance and nicotine addiction, and potentially absolving tobacco companies of responsibility.

Finally, some participants indicated that there were major structural challenges involved. An agency which is government-owned but truly independent was a challenging idea and would take years to establish. Some participants felt much more detailed policy work was needed on the practicalities of the implementation of this model. Another Public Health Physician thought this model was too complex ("*This is written for people who understand policy"*) to communicate in its current formulation and needed to be simplified as to what it means in concrete terms for smokers and non-smokers.

### Action 1: Tobacco companies bid for reducing quotas

Several participants favoured this approach, seeing it as innovative, thoughtful and possible. Some saw it as a way to achieve the endgame, with tobacco gradually becoming invisible. The gradualness of the change was seen positively by making it politically and socially acceptable ("*less controversial"*), and offering a transition period before tobacco companies cease operating in the New Zealand market. Some made analogies with fishing quotas and carbon emissions trading, and previous restrictions to alcohol supply in New Zealand.

Other advantages cited were that the process of bidding for quotas would result in pitting tobacco companies against each other. Participants thought that the price of tobacco would go up as a result of the declining quota - a desirable result from a tobacco control perspective.

However, some participants found it difficult to see how the system would work, especially in relation to market forces and price, and the practicalities of supply at the population and individual level. One participant noted that this approach would need to be accompanied by strong activity to reduce demand (to match reducing supply), while another thought that individual smokers would need to be assigned a quota as well as the tobacco companies. This would require a verification system at purchase, and could create practical difficulties. Concerns about possible interruptions to supply were raised. These could be problematic, particularly for the most heavily addicted smokers.

*"Where it will cause freak-out is when the dairies (small local general stores) run out at the end of the month or whenever the release cycle is - you'll have the whole population going cold turkey." *- Public Health Physician

Others suggested a black market might develop, driven by the high price in legally available tobacco, and strong enforcement would be needed to control this. Problems were also foreseen on the basis that tobacco is a legally traded product. Quotas might risk challenges under the World Trade Organization rules. There were concerns about the impact of price rises on poor smokers, and resulting adverse media publicity. Making sure smokers had access to cessation support was seen as essential if the supply of tobacco was progressively restricted.

### Action 2: Penalties for tobacco companies, to promote rapid smoking reductions

Some participants were intrigued and fascinated by this idea and thought it a logical approach in line with the 'polluter pays' principle. However, overall there was less support for this intervention. Participants foresaw many negatives, particularly about practical issues of implementation. For example, given that there are several independent companies supplying the New Zealand market, it was difficult to see how they would be held collectively responsible, and how the share of penalties would be apportioned between companies. Participants thought that there was potential for this approach to encourage black market supply and gaming by companies, and noted that penalties would have to be very high to offset the profits from sales and deter companies. Potential legal problems were also identified, for example: companies may not be easily held accountable in law for individuals' behaviour or for reducing sales of a legal product; and if there is smuggling or a black market, companies could reasonably argue that smoking prevalence and consumption was not fully under their control. As a result of these concerns, participants thought that litigation and enforcement costs might be prohibitive.

One participant thought a major conceptual barrier was the ingrained nature of market theory in New Zealand. Participants felt that the end-point of this approach was unclear - for example, would tobacco be phased out and become illegal? Finally, some participants thought that penalty payments would need to be allocated to the health system to avoid the system being seen as a government revenue-gathering exercise.

### Action 3: New laws make it easy to take tobacco companies to court - and win

Changing laws to make it substantially easier for individuals, groups or the government to take tobacco companies to court for selling a harmful product was seen as a useful mechanism in tobacco control, with the advantage of moving the focus from the government to the courts and judiciary. It was seen by some as less radical and hence more 'doable' than other approaches, and could help counter 'nanny state' accusations against tobacco control efforts by ensuring that power and control are delegated to individuals. Participants were aware of major tobacco industry Court settlements and other successful outcomes in USA Courts.

However, overall there was less support for this action than for the other approaches. Several thought New Zealanders were insufficiently litigious for this measure to succeed, and there was a general reluctance to move towards a more litigious society. The approach was viewed (negatively) as creating *"a lawyers' heaven" *by one participant.

*"[I] worry about creating a litigious culture in New Zealand - you could start with tobacco because everyone agrees it's harmful, but... where does that principle take you? - anything perceived by someone else to be harmful becomes open to litigation" *- Policy official

In addition it was seen as a high-risk approach as there was great potential to lose in the Courts, especially given the tobacco industry's resources. Some expressed little faith in the court and justice system, citing the failure to enforce existing laws, such as selling tobacco to underage consumers, or felt that the Courts do not properly understand tobacco issues. Others saw successful US precedents as having little relevance, as the USA tort system differs from New Zealand's. There were concerns raised about the slowness and expense of using the Courts, the expertise of the industry in thwarting legal approaches, the fragmented and disorganised nature of relying on multiple individual Court cases, and the poor cost-effectiveness of using public resources in legal battles against the industry. Some questioned who would pay for the litigation.

*"...the companies have a massive incentive to pour immense resources into those [Court] cases... [it is] barking up the wrong tree to dedicate public resource to that" - *Policy official

Finally, the approach was thought by some participants to be too indirect, and another noted that that while it may make things harder for the tobacco companies, it was unlikely to be a rapid method to achieve endgame goals. Government regulation was seen as a more direct and more effective approach.

***"**If there are things we want to prohibit, we should do that directly through government policy and regulation rather than asking the Courts to make judgements" - *Policy official

*"..if tobacco companies are including chemicals that make tobacco more addictive and sweeter tasting ... rather than use Courts ....[The] Government could just decide they only want ... less harmful cigarettes in New Zealand." *- Policy official

### Other general themes

#### Nanny state and anti-bureaucracy political climate

Many participants noted that the current political environment and public sentiment in New Zealand was unfavourable to measures which could be seen or portrayed as increasing government control over individuals' lives, or resulted in the creation of new bureaucracies. Therefore a commonly stated view was that the proposed models and actions would be difficult to achieve politically, particularly creating a new organisation such as the Nicotine Authority or Tobacco Supply Agency.

*"How you are going to sell it politically in a climate where 'nanny-state' is poison?" - *Journalist

"...[it] *buys into the whole 'nanny state' thing - enormous backlash - here's yet another government authority telling us what to do." - Public Health Physician*

#### Preferences for using existing structures and mechanisms

Some participants favoured strengthening existing governmental tobacco control arrangements, such as the team within the Ministry of Health, and opposed establishing new stand-alone agencies. One reason was that setting up new agencies could take years. Another was that small stand-alone agencies may be less able to carry out complex regulatory functions than a government department which had the necessary critical mass, skills, expertise and resources - particularly in the face of skilful opposition from the tobacco industry. Finally, some participants thought recent experience suggested that new agencies were vulnerable to attack and disestablishment.

*"The history of regulatory bodies is that they've tended to come and go. When the going gets tough someone disestablishes them and takes the regulatory function back centrally" *- Policy official

Some simply preferred evolutionary change:

*"Philosophically... I am opposed to structural change... all my experience tells me you lose three or four years... setting up new entities, getting them going...; and so my approach ...is to use what you've got and push that harder" *- Policy official.

#### Framing of tobacco products and endgames

The importance of how tobacco products and tobacco control efforts are framed was a recurring theme. For example, some participants felt that many people in New Zealand thought that the battle against tobacco was largely over, particularly among older, more highly educated people, including policy and media workers. The reasons given included that smoking is much less visible and less socially acceptable, and that many people have become blasé about smoking as a significant health concern.

*"If smoking is off their radar screen in their everyday lives, it ... is harder to engage them in the idea that they need to do more about it" *- Journalist

Some participants noted that in order to 'sell' endgame solutions or structural reforms, the extent of the public health problem had to be clearly explained to create a clear rationale for action *("sell the problem before you sell the solution")*. This was described as establishing the '*burning platform*'.

One participant suggested that how tobacco products are framed dictates how they are treated by government and agencies of government, by the legal system and by society in general; and determines what is politically possible and acceptable. This participant thought the dominant framing of tobacco products was as a somewhat risky but legal commodity and as a tax source, making governments reluctant to intervene to control supply. (S)he argued that to overcome 'nanny state' accusations and make endgame solutions acceptable, tobacco products would need to be framed and viewed by society and policy-makers as highly hazardous (e.g. as a *'poison'*) to individuals and society, i.e. in the same way that hard drugs are generally viewed. Tobacco products would then be seen as unsuitable for normal commercial activity, and bolder and more rigorous measures to reduce tobacco use would be justified. Another participant noted that tobacco control needed to be framed like work on other public health hazards such as infectious diseases control, in order to justify urgent and concerted action.

The way major endgame interventions are framed was also seen as important in the communication of such approaches. For example, one view was that the benefits of tobacco control needed to be seen through a broader lens than that of public health, and be seen as part of society's greater goals for well-being and prosperity. The importance of linking the endgame interventions to the achievement of a credible and desirable vision was emphasised by some. Other participants noted that the use of analogies would be important to justify and explain the need for more rigorous measures. For example, the approach to tobacco could be compared and contrasted with 'party pills' (recently banned in New Zealand) and illegal drugs (for which supply-side measures are the norm). A point in favour of tobacco endgame solutions that some participants noted was that social attitudes have changed, that smoking is much less socially acceptable and there is no public sympathy for the tobacco companies. One participant argued that once the 'tipping point' was reached, politicians would likely jump on the endgame bandwagon to associate themselves with a winning issue.

## Discussion

We found that a tobacco-free vision was widely welcomed by public health practitioners, policy-makers and journalists. There was also general support for a greater focus on supply-side measures. The five proposed 'endgame' approaches were viewed as interesting, but there were widely differing views about the desirability, feasibility and likely effectiveness of each. There was a general sense that supply-side focused measures would be difficult to achieve politically in the current New Zealand setting, and numerous barriers were advanced.

Supply-side approaches were also viewed with caution by many participants. There may be many reasons for this caution including: novelty and unfamiliarity of the approaches; individual ideological or political views; the absence of current examples and evidence for successful supply-side focused approaches; a tendency to stick to known approaches; a preference for incremental, evolutionary approaches over radical and rapid change; 'market thinking' being ingrained, widespread and pervasive; awareness that an overt attack on market thinking and values is difficult and daring; and awareness that market agenda-setters have successfully framed attempts to regulate aspects of life where the market operates as illegitimate, unjustified interference by a 'nanny state'.

The extent of 'market thinking' was an echo of Borland's view in relation to the regulated market model that:

*'For countries like Australia, with strong commitments to free markets, there are challenges of rethinking how the model actually fits within a system that is designed to encourage competition and innovation' *[38].

A key finding was the importance of framing. The findings suggest that if tobacco is seen as just another legally traded commodity in the marketplace, supply-side interventions make little sense. But when tobacco is viewed as a highly hazardous and addictive product targeted at children, then regulation by the state and radical supply-side measures may be viewed as legitimate, desirable or even required in order to protect children and public health.

### Strengths and limitations of the research

This was a qualitative, exploratory research project, intended to open up a new field of study. As far as we are aware this is the first study which has explored reactions of key practitioners, opinion leaders and decision-makers to radical supply-focused endgame solutions for the tobacco epidemic. We successfully tested supply-side focused tobacco control approaches which have been proposed in the literature with informed and articulate research participants. Our impression of the interviews and focus groups was that the participants, though they had little or no previous awareness of such approaches, were highly engaged with the topic and as a group provided an incisive and well-informed commentary on the endgame approaches discussed. Comments from key informants provided insights into initial responses to supply-side approaches and how they might work in practice. We identified a range of key barriers, misunderstandings and difficulties which could inform communication strategies for endgame ideas.

An important limitation is that the findings may be somewhat context specific to New Zealand in 2008, and may not be generalisable to other populations and settings. Also, the discussions were of new and unfamiliar ideas and occurred somewhat in the abstract, as at the time of data collection none of these ideas had received any substantial exposure in the media or public discourse. Nevertheless, New Zealand shares many similarities with other English-speaking jurisdictions and discourses revealed (e.g. the 'nanny-state') occur in other countries [43,44]. Finally, this was a qualitative study with a small number of participants from highly selected sectors of society. The results may therefore not be generalisable. Similar studies would be needed to understand the views of the wider public, including smokers, and other key stakeholders potentially affected by different endgame policy options.

### Further research and policy analysis

Further research is suggested; in particular into exploring what underpins the doubts and reservations participants expressed and how these might be addressed, along with further policy analysis and development of the models and actions. Such work may help determine the degree to which these endgame strategies are likely to be effective and politically and logistically feasible to implement.

Specifically, policy analysis, research and economic modelling are needed on the relative costs and benefits of different models/actions versus the status quo, or expanded tobacco control within existing structures. Specific work is also needed on trade law requirements; taxation; framing of tobacco; smuggling and growing tobacco; implementation of particular models; legal, administrative, commercial and equity effects and requirements, and public information and communication approaches. Lessons may also be learnt from closer studies of policy-makers and public attitudes to other endgames (e.g., eradicating infectious diseases such as poliomyelitis, or phase-outs of other hazardous products such as leaded petrol, chlorofluorocarbons and asbestos).

### Implications for practice

The findings suggest approaches for advocacy efforts for the endgame. These include: better establishing the extent and urgency of the public health problem due to smoking; developing a credible tobacco-free vision; the importance of framing of tobacco products as extremely hazardous to society and a threat to children's well-being; developing a counter to the 'nanny state' discourse and legitimising the state's role in protecting citizens from unscrupulous business practices and dangerous products; and using simple and clear analogies to justify the use of supply-side approaches.

## Conclusions

The findings suggest that supply-side focused tobacco control interventions and bold endgame solutions are worth exploring further. The findings suggest that a significant change in the public framing of tobacco may be a prerequisite for implementing endgame solutions. Further policy analysis, research and public discussion are needed to investigate and further develop endgame approaches. This work is important, as these ideas could help eliminate the enormous burden of ill health, preventable death and health inequalities caused by the tobacco epidemic.

## Abbreviations

RE: Richard Edwards; MR: Marie Russell.

## Competing interests

The authors declare that they have no competing interests.

## Authors' contributions

RE was the principal investigator for the Daring to Dream research project and wrote the first and subsequent draft of this manuscript. MR carried out the bulk of the day to day work on the project, including organising and carrying out data collection and analysis. NW, GT and HG contributed to the initial conceptualisation of the research, and to the design and implementation of the study at every stage. All authors took part in discussions about the scope and content of this paper, and contributed comments and text to each draft. All contributing authors have read and approved the final manuscript.

## Pre-publication history

The pre-publication history for this paper can be accessed here:

http://www.biomedcentral.com/1471-2458/11/580/prepub
